# Correlation between anthropometric measurements and graft size in anterior cruciate ligament reconstruction: a systematic review and meta-analysis

**DOI:** 10.1007/s00590-023-03712-w

**Published:** 2023-09-06

**Authors:** Loay A. Salman, Isam Sami Moghamis, Ashraf T. Hatnouly, Harman Khatkar, Mohanad Mutasem Alebbini, Abdallah Al-Ani, Shamsi Hameed, Mohamed AlAteeq Aldosari

**Affiliations:** 1 Orthopedic Surgery Department, Hamad General Hospital, Hamad Medical Corporation, PO Box 3050, Doha, Qatar; 2 Royal London Hospital, Whitechapel, London, UK; 3 Office of Scientific Affairs and Research, King Hussein Cancer Center, Amman, Jordan

**Keywords:** Anterior cruciate ligament, Knee, Reconstruction, Anthropometric, Correlation

## Abstract

**Purpose:**

This systematic review and meta-analysis aimed to investigate the correlation between anthropometric measurements and graft size in anterior cruciate ligament (ACL) reconstruction.

**Methods:**

A systematic search of Ovid MEDLINE, Embase, and Cochrane Library databases was conducted for observational studies published until March 2023 that reported the relationship between anthropometric data [height, weight, body mass index (BMI), age, gender, thigh length, and circumference] and ACL graft size. Correlation coefficients (COR) and their associated 95% confidence intervals were used as the primary effect size. This review was conducted in line with PRISMA guidelines.

**Results:**

A total of 42 observational studies involving 7110 patients were included, with a mean age of 29.8 years. Statistically significant, moderately positive correlations were found between graft size and height (COR: 0.49; 95% CI: 0.41–0.57; *p*-value: < 0.001), weight (COR: 0.38; 95% CI: 0.31–0.44; *p*-value: < 0.001), thigh circumference (COR: 0.40; 95% CI: 0.19–0.58; *p*-value: < 0.001), and thigh length (COR: 0.35; 95% CI: 0.18–0.50; *p*-value: < 0.001). However, age and gender were insignificantly correlated with graft size (*p*-value: NS). A subanalysis based on graft type showed a significant positive correlation between height and graft diameter, which was more significant in the peroneus tendon than in hamstring grafts (COR: 0.76 vs. 0.45; *p*-value: 0.020).

**Conclusion:**

This study demonstrated a moderate positive correlation between anthropometric measurements (height, weight, thigh circumference, and length) and ACL graft size, along with a weak positive correlation with BMI. Age and gender showed no significant correlation. These findings support the predictability and selection of ACL graft size based on pre-operative patient anthropometric data.

**Level of evidence:**

Level of Evidence: IV.

**PROSPERO registration number**: CRD42023416044.

**Supplementary Information:**

The online version contains supplementary material available at 10.1007/s00590-023-03712-w.

## Introduction

Anterior cruciate ligament (ACL) injury is a common knee injury with an incidence of up to 78 per 100,000 person-years [1]. Surgical treatment is often required to restore knee biomechanics and function. Several autograft options are available for ACL reconstruction, such as bone-patellar tendon-bone (BTB), hamstring tendon (HT), quadriceps tendon (QUAD) and peroneal tendon (PLT) [2, 3], while the popularity of hamstring tendon grafts has risen due to their biomechanical stability, low donor-site morbidity and improved fixation methods [4, 5]; however, the success of the surgery is closely related to graft size, and inadequate graft size is associated with high failure and re-rupture rates.

Consequently, identifying patients with inadequate graft size has become essential for appropriate pre-operative decision-making and arrangement of alternative grafts source. Anthropometric measurements related to demographic and radiological parameters have been proposed to predict hamstring tendon graft size [6–9]. Several studies investigated the correlation between these measurements and graft size, but the results have been inconsistent [10–12].

Therefore, this systematic review and meta-analysis aimed to synthesise the best available evidence and comprehensively review the relationship between various anthropometric measures and graft size in ACL reconstruction surgery. This study also aimed to identify the most reliable predictors of tendon graft size to improve pre-operative planning and enhance patient outcomes.

## Methods

This systematic review was conducted in line with the Preferred Reporting Items for Systematic Reviews and Meta-Analyses (PRISMA) guidelines [13]. A protocol registration was completed in advance on the International Prospective Register of Systematic Reviews (PROSPERO) with the registration number: CRD42023416044.

### Search strategy

Ovid MEDLINE, Embase, and Cochrane Library databases were searched from inception until March 2023 with the following keywords and their derivatives: Anterior cruciate ligament, ACL, anthropometric measurements, height, weight, body mass index, age, gender, thigh length, and circumference. Search results were screened against the eligibility criteria by two authors independently based on the title and/or abstract. Conflicts were resolved via a discrepancy meeting with a third senior author, if needed.

### Outcomes of interest

Correlation between height and graft size was the primary outcome. Correlation between graft size and other anthropometric measures including weight, BMI, gender, thigh length and circumference, and graft types were used as secondary outcomes of interest. Moreover, correlation is described as a measure of association between variables either in the same (positive correlation) or in the opposite (negative correlation) direction and range between − 1 and + 1 [14].

### Eligibility criteria

Studies were considered eligible if they satisfied the following criteria: (1) all original observational studies reporting correlation between anthropometric measurements (height, weight, BMI, gender, age, thigh circumference, and length) and actual intraoperative graft size in adult population, (2) all types of ACL grafts (Hamstrings, Peroneus longus, BPB, and Quadriceps,), and (3) published in the English language.

Exclusion criteria included (1) studies not correlating anthropometric measurements with actual intraoperative graft size, (2) studies correlating anthropometric measurements or graft size with MRI or other means, (3) studies with incomplete or unextractable data for review, and (4) review articles, preclinical, cadaveric and anatomical studies, and case reports.

### Data extraction and items

Two independent reviewers used a pre-designed data collection sheet in Microsoft Excel to extract data. The extracted demographic data included the first authors’ surnames, study year, design and country, number of participants and knees, population type (adult vs paediatrics), graft type, the mean age of patients, gender, mean height, weight, BMI, thigh length and circumference, level of activity, correlations reported for each variable, statistical tests, and conclusions.

### Qualitative assessment (risk of bias)

Two authors assessed the methodological quality of the included studies using the Methodological Index for Non-Randomized Studies (MINORS) assessment tool, which comprise eight key items, with a global ideal score of 16 for non-comparative studies [15]. A higher overall score indicates a lower risk of bias; a score of 8 or less corresponds to a high risk of bias.

### Statistical analysis

A meta-analysis of the eligible studies using R (version 4.0.2, R Core Team, Vienna, Austria, 2020) was conducted using the meta package (i.e. forest_meta and metacor). Correlation coefficients (COR) and their associated 95% confidence intervals were presented as the main effect size. For studies that reported beta regression values instead of Pearson’s r, the latter was estimated using the equation *r* = 0.98ß + 0.5*λ* published by Peterson and Brown [16]. Strength of the resultant effect sizes was interpreted per the criteria set by Cohen (*x* < 0.1, weak; 0.3 < *x* < 0.5, moderate; *x* > 0.5, strong) [17]. Heterogeneity among effect sizes was evaluated using the *I*-squared statistic. Definitions for heterogeneity were adapted from the Cochrane handbook (< 25%, mild; 25–50%, moderate; > 50%, severe). Due to the high heterogeneity for the dichotomous variables, a random-effects model was utilised. Both a funnel plot and Egger’s test of asymmetry were utilised to assess publication bias.

## Results

### Study selection

**Fig. 1:**
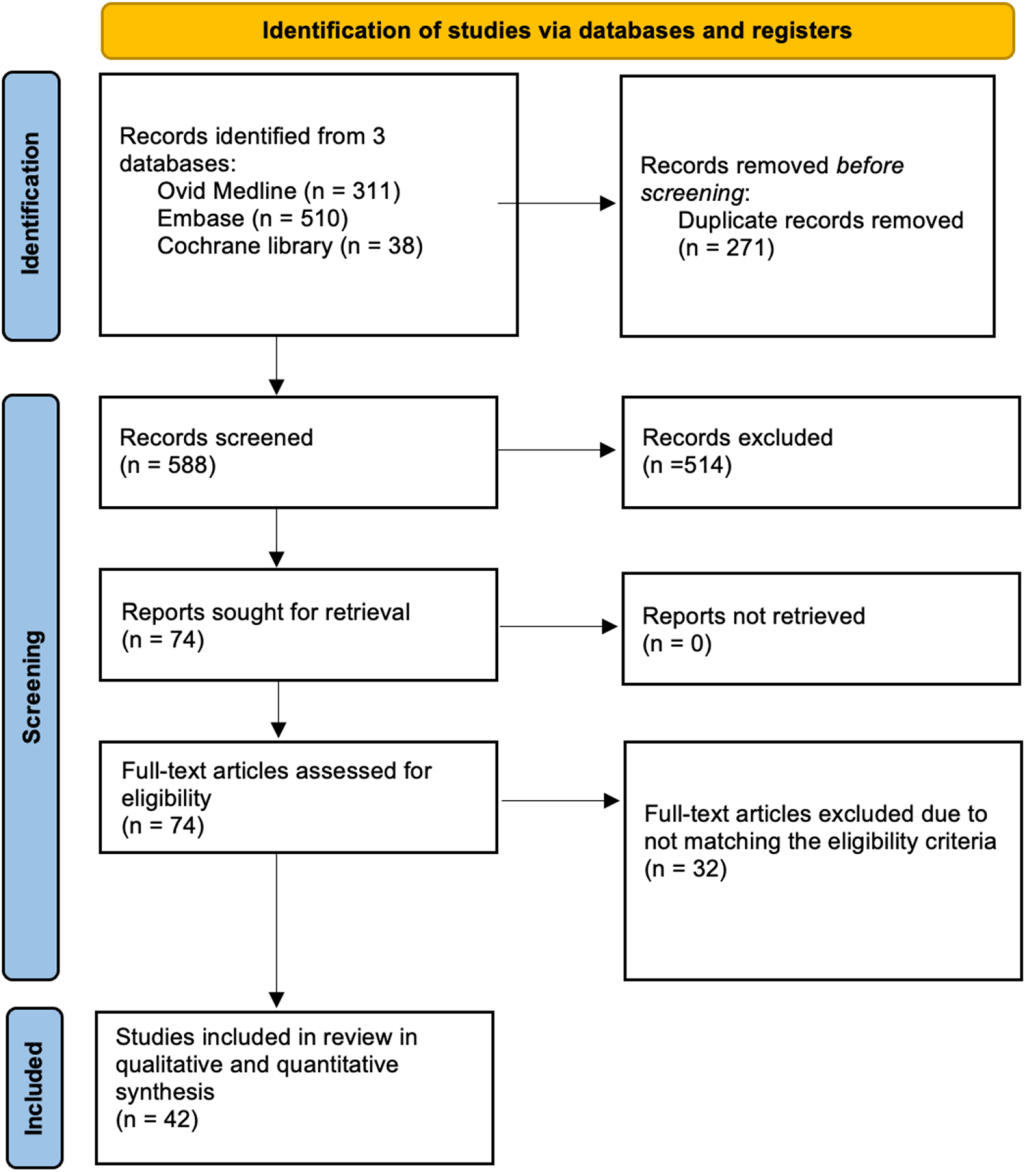
PRISMA flow diagram of record identification, screening and selection in meta-analysis

Searching the databases yielded 859 articles, and after removing 271 duplicates, 588 records were screened by title and abstracts, of which 514 were excluded. A total of 74 papers were eligible for a full-text review. As a result, 42 studies met the eligibility criteria and were included in the qualitative and quantitative synthesis. The PRISMA flowchart is displayed in Fig. 1.

### Quality assessment [risk of bias and level of evidence (LoE)]

Based on the OCEBM criteria [18], 21 studies were level 2, 15 were level 3, and 6 were level 4 (Table 1), with an overall grade B of recommendation assigned to the review [19]. The MINORS criteria scores of all 42 observational studies ranged from 10 to 15, with an average of 12.71 ± 1.29 (Out of 16), indicating a low overall risk of bias. A summary of the qualitative assessment, according to the MINORS criteria, is shown in the Supplementary material.

**Table 1:** A summary of baseline study characteristics

Study	Design, LoE	Country	Population	No. of patients	Graft type (strands)	Graft source
2007 Brown [6]	Cohort, II	USA	Adult	414	BTB	Allograft
2007 Tuman [7]	Cohort, II	USA	Adult	106	HT	Autograft
2008 Treme [5]	Cohort, II	USA	Adult	50	HT	Autograft
2012 Chan [30]	Series, IV	USA	Adult	20	HT	Autograft
2012 Reboonlap [31]	Cross-sectional, III	Thailand	Adult	74	HT	Autograft
2012 Stergios [32]	Retrospective, III	Greece	Adult	61	HT	Autograft
2012 Xie [33]	Cohort, II	China	Adult	235	HT	Autograft
2013 Celiktas [34]	Cohort, II	Turkey	Adult	164	HT	Autograft
2013 Challa [4]	Cohort, II	India	Adult	41	HT	Autograft
2013 Park [35]	Series, IV	South Korea	Adult	296	HT	Autograft
2013 Thomas [9]	Cohort, II	UK	Adult	121	HT	Autograft
2014 Schwartzberg [10]	Cohort, II	USA	Adult	100	HT	Autograft
2015 Nuelle [36]	Series, IV	USA	Adult	60	HT	Autograft
2016 Asif [37]	Retrospective, III	India	Adult	46	HT	Autograft
2016 Atbasi [38]	Retrospective, III	Turkey	Adult	126	HT	Autograft
2016 Goyal [39]	Cohort, II	India	Adult	160	HT	Autograft
2016 Ho [40]	Series, IV	Singapore	Adult	169	HT	Autograft
2016 Kivi [41]	Cross-sectional, III	Iran	Adult	178	HT	Autograft
2016 Pereira [11]	Retrospective, III	Brazil	Adult	64	HT	Autograft
2016 Sundararajan [42]	Cohort, II	India	Adult	108	HT	Autograft
2017 Chiba [43]	Cross-sectional, III	Japan	Adult	200	HT	Autograft
2017 Gupta [44]	Cohort, II	India	Adult	123	HT	Autograft
2017 Leiter [12]	Retrospective, III	Canada	Adult	109	HT	Autograft
2017 Vincent V.G. An [45]	Retrospective, III	Australia	NR	108	HT	
2018 Ramkumar [46]	Cross-sectional, III	USA	Adult	1681	HT	Autograft
2018 Song [47]	Retrospective, III	China	Adult	156	PLT	Autograft
2019 Heijboer [48]	Cohort, II	Netherlands	Adult	53	HT	Autograft
2019 Moghamis [8]	Mixed, III	Qatar	Adult	50	HT	Autograft
2019 Sakti [49]	Cohort, II	Indonesia	Adult	60	HT	Autograft
2020 Du-Hyun Ro [50]	Retrospective, III	Korea	Adult	54	HT	Autograft and allograft
2020 Goyal [51]	Cohort, II	India	Adult	95	QUAD	Autograft
2020 Jagadeesh [52]	Cohort, II	India	Adult	128	HT	Autograft
2020 Sakti [53]	Cohort, II	Indonesia	Adult	20	PLT	Autograft
2020 Thwin [54]	Cohort, II	Singapore	Adult	141	HT	Autograft
2021 Ertilav [55]	Retrospective, III	Turkey	Adult	53	PLT	Autograft
2021 Khan [56]	Retrospective, III	India	Adult	52	PLT	Autograft
2021 Kumar [57]	Retrospective, III	India	Adult	73	HT	Autograft
2021 Singhal [58]	Cohort, II	India	Adult	280	HT	Autograft
2022 Harshith [59]	Cohort, II	India	Adult	35	HT	Autograft
2022 Huang [60]	Cohort, II	China	Adult	24	HT	Autograft
2022 Mishra [61]	Series, IV	India	NR	256	HT	Autograft
2023 Movahedinia [62]	Cohort, II	Iran	Adult	42	HT	Autograft

Study	Age (*Y*)	Height (cm)	Weight (Kg)	BMI (kg/m^2^)	Gender (*M*/*F*)	Thigh length (cm)	Thigh circumference (cm)	Sports/Activity level
2007 Brown [6]	45.8 ± 17.4	172 ± 11.4	74 ± 15.4	NR	1.10	NR	NR	NR
2007 Tuman [7]	32.9 ± 14.1	172.4 ± 9.4	75.4 ± 14.9	25.4 ± 4.8	0.92	NR	NR	NR
2008 Treme [5]	31.6 ± 13.6	170.9 ± 10.5	78 ± 18.4	28.4 ± 4.7	1.37	51.8 ± 4.9	47.0 ± 4.9	Tegner score 6.4 ± 2.0
2012 Chan [30]	28.14	172.1	75.0	24.54	1.50	NR	NR	NR
2012 Reboonlap [31]	29.2 ± 9.0	171.9 ± 6.9	71.2 ± 10.4	24.0 ± 2.8	0.00	52.7 ± 3.8	47.4 ± 3.8	NR
2012 Stergios [32]	27.0 ± 7.7	176.2 ± 8.3	77.8 ± 14.1	24.9 ± 3.5	2.81	NR	NR	NR
2012 Xie [33]	28.1 ± 10	171.9 ± 7.9	71.0 ± 13.7	23.9 ± 3.5	2.45	NR	NR	Tegner score 6.15 ± 0.8
2013 Celiktas [34]	29.23	179.2 ± 5.3	82.5 ± 8.8	25.7 ± 2.3	0.00	NR	51.0 ± 4.7	NR
2013 Challa [4]	27.9 ± 8.9	170.8 ± 5.3	66.5 ± 7.1	22.7 ± 2.8	4.85	NR	NR	NR
2013 Park [35]	29.8 ± 10.7	171.3 ± 7.6	72.1 ± 12.2	24.5 ± 3.3	3.84	NR	NR	11% Athletes
2013 Thomas [9]	31.9	177	84.90	26.90	8.31	NR	NR	NR
2014 Schwartzberg [10]	NR	NR	NR	NR	NR	NR	NR	NR
2015 Nuelle [36]	25.3 ± 8.9	176.4 ± 10.6	79.4 ± 16.7	25.3 ± 3.9	1.5	NR	NR	All athletes
2016 Asif [37]	29.4 ± 10.2	172.6 ± 4.6	70.9 ± 11.5	23.8 ± 3.7	22.00	NR	47.1 ± 5.0	NR
2016 Atbasi [38]	24.2 ± 4.6	176.3 ± 5.4	77.9 ± 8.1	25.1 ± 2.3	0.00	NR	NR	NR
2016 Goyal [39]	NR	169.1 ± 6.9	69.2 ± 11.7	24.1 ± 3.5	NR	51.5 ± 3.5	NR	NR
2016 Ho [40]	25.5	171.3	73.54	25.25	5.03	NR	NR	NR
2016 Kivi [41]	29.8 ± 9.9	174.8 ± 7.8	76.4 ± 12.7	24.9 ± 3.5	1.96	NR	NR	NR
2016 Pereira [11]	31.8 ± 8.2	177 ± 8.0	82.4 ± 12.9	26.1 ± 3.7	15.00	NR	NR	NR
2016 Sundararajan [42]	33.0 ± 9.5	167.7 ± 9.9	72.4 ± 12.4	25.7 ± 3.6	4.40	51.5 ± 4.1	NR	NR
2017 Chiba [43]	25.6 ± 13	165.6 ± 8	63.5 ± 11.9	23.1 ± 3.5	0.77	NR	NR	Tegner score 6.4 ± 1.9
2017 Gupta [44]	28.4 ± 8.8	173.3 ± 7.3	75.0 ± 11.3	NR	7.20	49.4 ± 3.6	48.2 ± 3.8	NR
2017 Leiter [12]	27.8 ± 11.4	173.0 ± 12.0	80.6 ± 19.6	26.9 ± 5.7	1.82	NR	NR	NR
2017 Vincent V.G. An [45]	30.7 ± 13.9	172.9 ± 9.6	NR	NR	1.47	NR	NR	NR
2018 Ramkumar [46]	28.7 ± 11.8	172.7 ± 10.0	80.1 ± 18.6	26.8 ± 5.1	1.45	NR	NR	NR
2018 Song [47]	29.5 ± 8.1	174.1 ± 8.6	76.2 ± 13.2	25.0 ± 3.4	1.44	NR	NR	NR
2019 Heijboer [48]	25	178.0 ± 8.9	78.2 ± 14.0	NR	3.10	NR	46 ± 3.8	Tegner score 9(7.3–9)
2019 Moghamis [8]	29 ± 7	174.0 ± 8.0	82.2 ± 11.2	27.0 ± 3.5	0.00	46.6 ± 2.7	50.7 ± 3.8	NR
2019 Sakti [49]	27.2 ± 7.5	167.7 ± 7.1	71.9 ± 15.7	25.4 ± 4.7	5.66	38.8 ± 3.8	45.8 ± 6.9	NR
2020 Du-Hyun Ro [50]	28.2 ± 9.2	169.8	66.8	23.57	1.45	NR	NR	NR
2020 Goyal [51]	30.2 ± 8.7	168.1 ± 7.3	72.2 ± 11.2	25.6 ± 3.7	NR	46.9 ± 4.1	47.5 ± 5.9	Tegner score 4
2020 Jagadeesh [52]	30.8 ± 10.1	167.4 ± 6.3	66.5 ± 7.9	23.7 ± 2.6	0.00	50.0 ± 2.4	NR	NR
2020 Sakti [53]	29.8	168.1 ± 8.2	71.2 ± 13.1	25.0 ± 3.1	5.66	NR	NR	NR
2020 Thwin [54]	24.77	171.1	72.78	24.69	4.42	NR	NR	NR
2021 Ertilav [55]	29.2 ± 7.7	170.0 ± 10.0	76.0 ± 12.6	25.9 ± 2.6	2.00	NR	NR	NR
2021 Khan [56]	28.2 ± 7.4	172.7 ± 2.8	75.6 ± 3.4	25.3 ± 0.9	7.66	NR	NR	NR
2021 Kumar [57]	33.7 ± 11.2	173.1 ± 5.3	71.2 ± 13.1	23.7 ± 3.9	0.00	NR	50.4 ± 6.8	NR
2021 Singhal[58]	28.6 ± 8.7	1.69 ± 0.1	75.2 ± 14.2	26.3 ± 4.6	4.18	NR	NR	NR
2022 Harshith [59]	33.2 ± 6.9	166.4 ± 9.6	70.1 ± 9.4	25.1 ± 4.5	6.00	49.3 ± 4.6	44.2 ± 5.0	NR
2022 Huang [60]	33.7 ± 8.4	NR	NR	NR	1.18	NR	NR	NR
2022 Mishra [61]	NR	NR	NR	NR	NR	NR	NR	NR
2023 Movahedinia [62]	32.8 ± 5.1	173.8 ± 5.6	77.1 ± 7.3	25.4 ± 2.0	3.2	NR	NR	NR

*LoE* level of evidence, *FU (Y)* follow-up in years, *HT* hamstring, *PLT* peroneus longus tendon, *BMI* body mass index

### Pooled study characteristics

A total of 42 studies satisfied the study’s eligibility criteria. Included reports spanned the years between 2007 and 2022. The majority of studies originated from India (27.9%) and USA (16.3%). Pooled number of participants for all studies was 7110 patients ranging from 20 to 1681 with a mean age of 29.8 years (24.2–45.8). Mean pooled height and weight for included participants were 172.7 (165.6–179.17) cm and 76.1 (63.5–84.9) kgs, respectively. Additionally, mean pooled BMI was 25.4 (22.7–28.4) kg/m^2^. Of the studies that reported gender stratifications, the majority were predominated by male patients (94.8%) with 6 studies including a cohort of only males (15.4%). Mean pooled thigh length and circumference were 49.4 (38.8–52.7) cm and 48.4 (44.2–51.0) cm, respectively. Hamstring grafts were the most prevalent among included studies (86.1%), followed by PLT (9.3%), QUAD (2.3%), and BTB (2.3%). Furthermore, mean pooled graft length and diameter for hamstring grafts were 261.5 (124.3–318.7) mm and 7.8 (4.7–9.0) mm, respectively. The graft length and diameter for the only study utilising QUAD grafts were 277 mm and 8.4 mm, respectively. Length and diameters for studies using the PLT and BTB grafts were not reported.

### Correlations between graft diameter and anthropometric measures

**Fig. 2:**
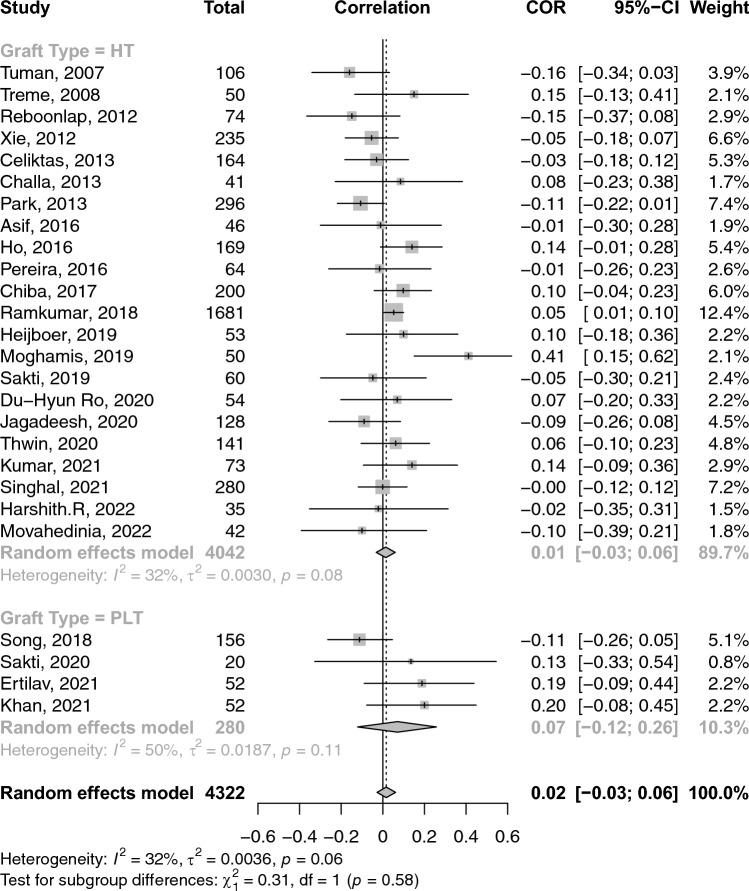
Forest plot of the overall pooled correlation between age and graft diameter. *COR* Correlation, *CI* confidence interval

**Fig. 3:**
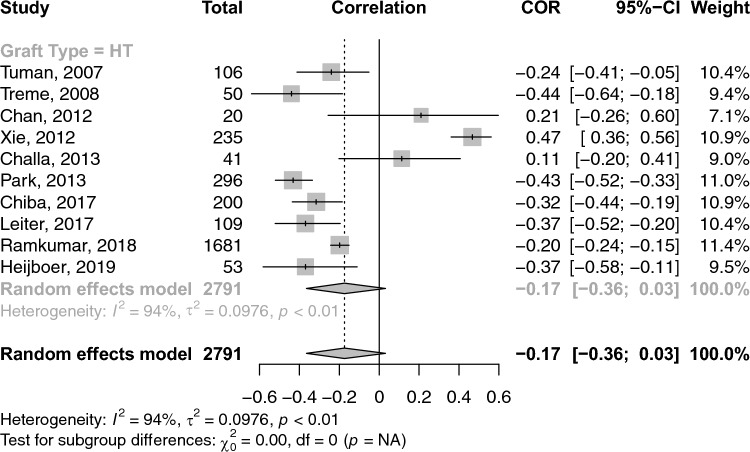
Forest plot of the overall pooled correlation between gender and graft diameter. *COR* Correlation, *CI* confidence interval

**Fig. 4:**
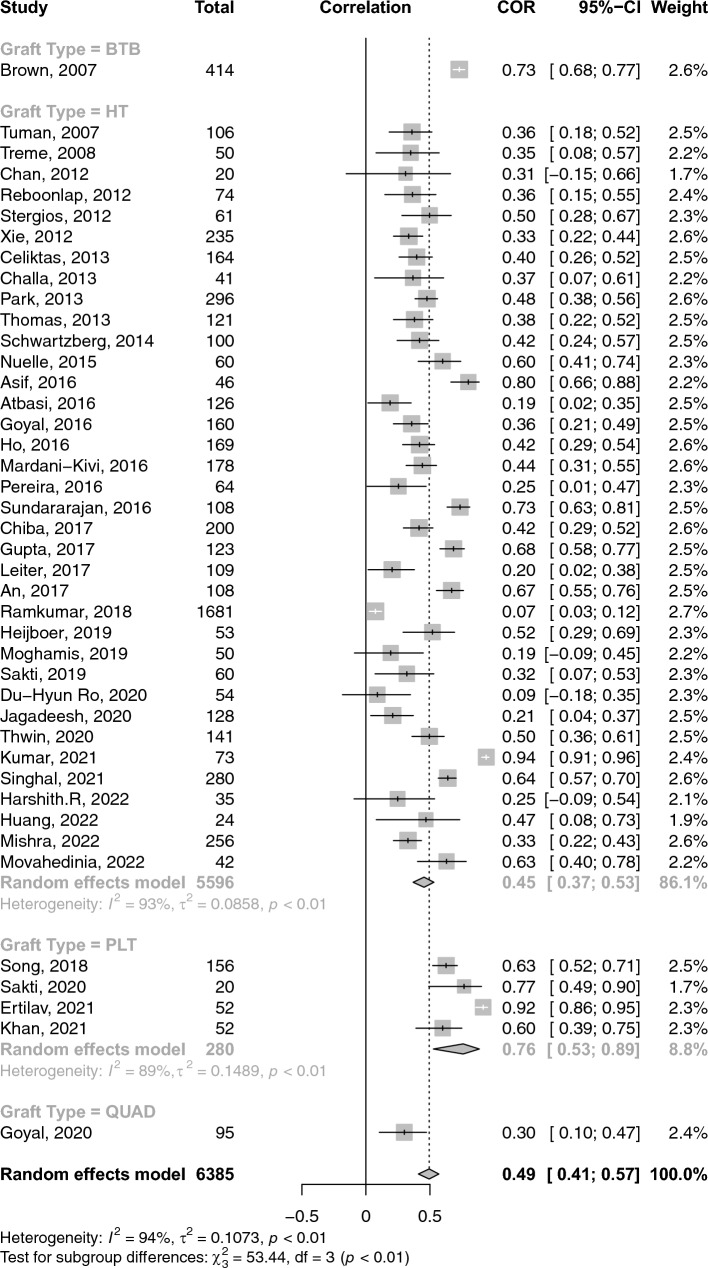
Forest plot of the overall pooled correlation between height and graft diameter. *COR* Correlation, *CI* confidence interval

**Fig. 5:**
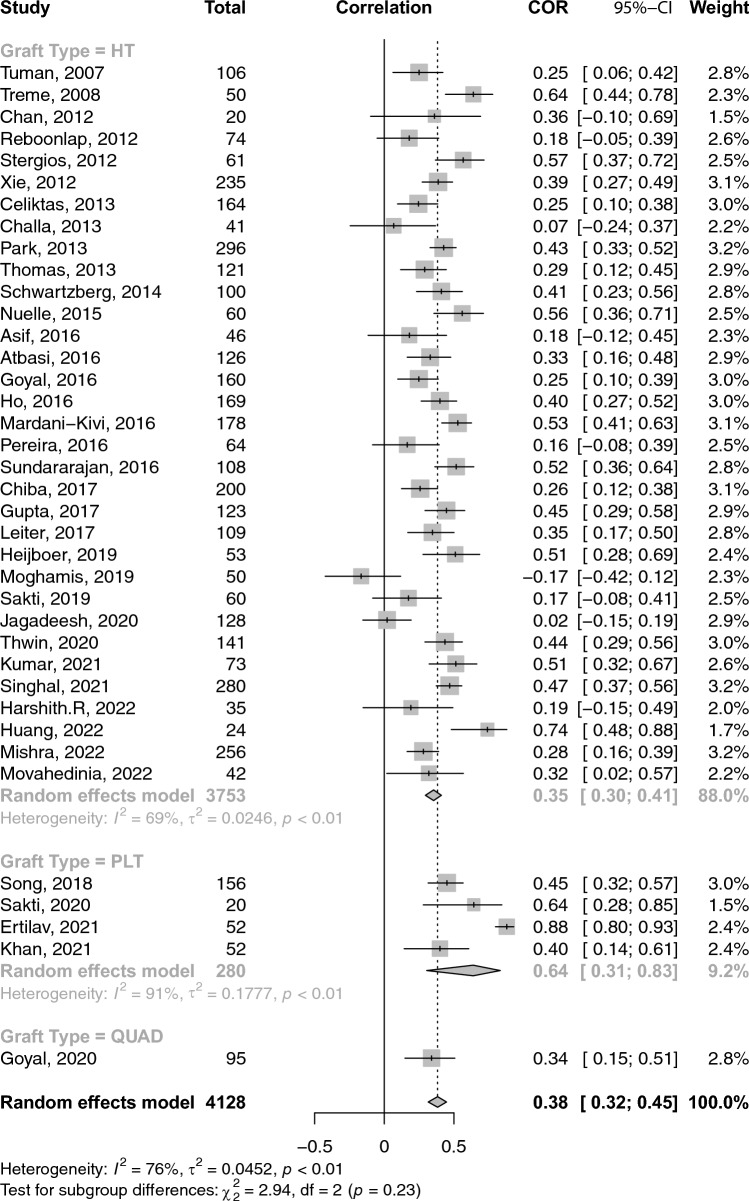
Forest plot of the overall pooled correlation between weight and graft diameter. *COR* Correlation, *CI* confidence interval

**Fig. 6:**
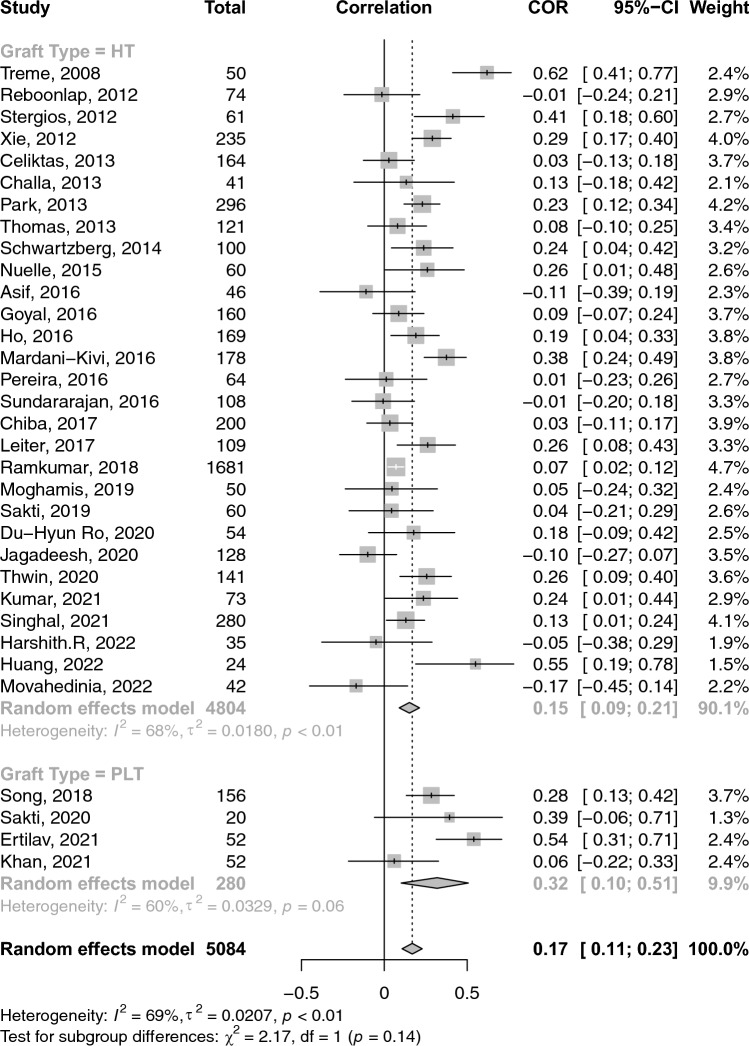
Forest plot of the overall pooled correlation between BMI and graft diameter. *COR* Correlation, *CI* confidence interval

**Fig. 7:**
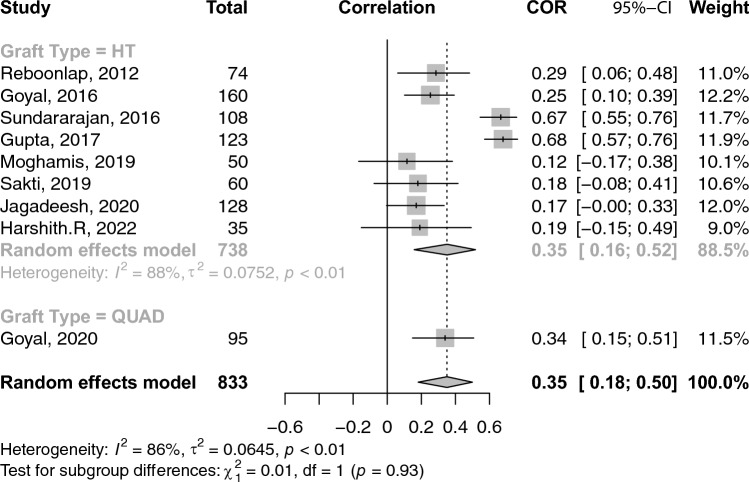
Forest plot of the overall pooled correlation between thigh length and graft diameter. *COR* Correlation, *CI* confidence interval

**Fig. 8:**
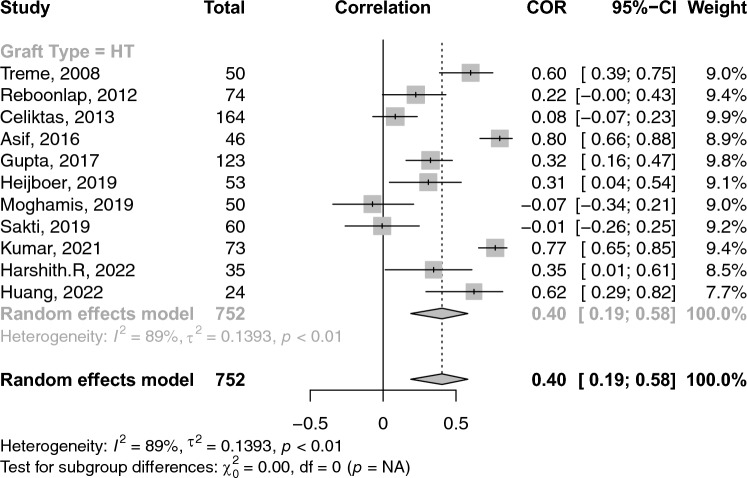
Forest plot of the overall pooled correlation between thigh circumference and graft diameter. *COR* Correlation, *CI* confidence interval

A total of 26 studies reported on the correlations between age and graft diameter. The pooled correlation between age and graft diameter was extremely small and insignificant (COR: 0.02; 95% CI: − 0.03–0.06; *p*-value: 0.462) (Fig. 2). With respect to gender and graft size, an insignificant weak negative (i.e. favouring males) correlation was observed (COR: − 0.17; 95% CI: − 0.36–− 0.03; *p*-value: 0.096) (Fig. 3). Height and weight correlated moderately with graft size (COR: 0.49; 95% CI: 0.41–0.57; *p*-value: < 0.001) and (COR: 0.38; 95% CI: 0.31–0.44; *p*-value: < 0.001), respectively (Figs. 4 and 5). Moreover, BMI correlated weakly yet positively with graft size (COR: 0.17; 95% CI: 0.11–0.23; *p*-value: < 0.001) (Fig. 6). Additionally, thigh length and circumference were moderately correlated with graft diameter (COR: 0.35; 95% CI: 0.18–0.50; *p*-value: < 0.001) and (COR: 0.40; 95% CI: 0.19–0.58; *p*-value: < 0.001), respectively (Figs. 7 and 8). A summary of the main correlation analysis is shown in Table 1.

### Subgroup analysis per graft type and region

When stratified by graft type**,** the correlation between age and graft diameter did not significantly differ between hamstring- and PLT-using studies (COR: 0.01 vs. 0.02; *p*-value: 0.580). Conversely, height was significantly more strongly correlated with graft diameter within PLT-using studies than their hamstring counterparts (COR: 0.76 vs. 0.45; *p*-value: 0.020). PLT-using studies demonstrated a strong correlation between weight and graft diameter compared to their hamstring-using counterparts; however, such difference was insignificant (COR: 0.64 vs. 0.35; *p*-value: 0.09). Similarly, differences in BMI correlation with graft diameter were statistically insignificant between PLT- and hamstring-using studies (COR: 0.32 vs. 0.15; *p*-value: 0.140). Stratification of correlations between anthropomorphic measures and graft diameter across different nations and graft types is provided in Tables 2 and 3.

**Table 2:** A summary of the primary correlation analysis of anthropometric measures and ACL graft size

Outcome	No. studies	No. patients	Correlation (*r*)	95% CI	Heterogenity *I*^2^ (%)	*p*-value
Age (Fig. 2)	26	4322	0.016	− 0.03–0.06	32.1	0.461
Gender (Fig. 3)	10	2791	− 0.173	− 0.36–− 0.03	94.2	0.096
Height (Fig. 4)	42	6385	0.494	0.41–0.56	94.0	< 0.001
Weight (Fig. 5)	38	4128	0.383	0.31–0.44	76.3	< 0.001
BMI (Fig. 6)	33	5084	0.168	0.11–0.23	68.8	< 0.001
Thigh length (Fig. 7)	9	833	0.351	0.18–0.50	86.1	< 0.001
Thigh circumference (Fig. 8)	11	752	0.403	0.19–0.58	89.1	< 0.001

*BMI* body mass index, *CI* confidence interval

**Table 3:** Subanalysis based on graft type comparing the correlation of anthropometric measures and graft size in Hamstring (HT) versus peroneus longus tendon (PLT) grafts

Outcome	No. studies	No. patients	Correlation (*r*)	Heterogeneity *I*^2^ (%)
HT				
Age	22	4042	0.14 (− 0.03–0.06)	31.9
Height	36	5596	0.45 (0.36–0.53)	92.6
Weight	33	3753	0.35 (0.29–0.41)	69.1
BMI	29	4804	0.15 (0.09–0.21)	67.6
PLT				
Age	4	280	0.07 (− 0.12–0.25)	49.7
Height	4	280	0.75 (0.53–0.88)	89.3
Weight	4	280	0.64 (0.31–0.83)	90.8
BMI	4	280	0.32 (0.10–0.51)	60.2

*BMI* body mass index

### Heterogeneity and publication bias

Significant heterogeneity was present across all pooled correlations ranging from 32.0 to 94.0%. Egger’s test indicated funnel plot asymmetry for only the studies reporting on correlation between height and graft diameter (*p* = 0.004). Funnel plots for all pooled correlations are included within the supplementary material (Table 4).

**Table 4:** Subanalysis based on region comparing the correlation of anthropometric measures and graft size in Hamstring (HT) versus peroneus longus tendon (PLT) grafts

Outcome	No. studies	No. patients	Correlation (*r*)	Heterogeneity *I*^2^ (%)
North America				
Age	3	1837	0.01 (− 0.14–0.16)	60.1
Height	8	2540	0.41 (0.22–0.56)	97.3
Weight	6	445	0.43 (0.29–0.54)	55.0
BMI	5	2000	0.28 (0.09–0.45)	84.7
Asia				
Age	17	2060	0.002 (− 0.05–0.05)	14.5
Height	23	2826	0.52 (0.40–0.62)	90.3
Weight	22	2772	0.36 (0.29–0.42)	66.1
BMI	20	2352	0.14 (0.07–0.19)	52.2
Europe				
Age	3	269	0.05 (− 0.09–0.18)	3.6
Height	6	577	0.54 (0.22–0.75)	92.8
Weight	6	577	0.51 (0.23–0.71)	91.0
BMI	4	398	0.26 (− 0.00–0.49)	83.0
Middle East				
Age	2	92	0.17 (− 0.34–0.60)	83.8
Height	3	270	0.43 (0.18–0.63)	68.7
Weight	3	270	0.25 (− 0.17–0.60)	90.7
BMI	3	270	0.11 (− 0.21–0.42)	84.0

*BMI* body mass index

## Discussion

This systematic review and meta-analysis represents the first large-scale quantitative analysis of anthropometric data in relation to ACLR. It may represent a starting point for evidence-based decisions relating to patient selection, graft size, and subsequent clinical outcome.

### Correlations between graft diameter and anthropomorphic measures

The correlation between age and graft diameter was deemed statistically insignificant. Clinically, this would be supported by evaluating the patient demographic undergoing ACLR. This would generally include the active adult population, in which muscular conditioning, development, and thus graft size would generally be considered comparable [20, 21]. Where this correlation may be clinically significant would be in the elderly population, where ACLR may not be so readily performed due to poor-quality graft availability as a result of age-related sarcopenia [22, 23].

The weak insignificant correlation favouring an association between male gender and graft size should be treated with caution within the context of this review. This is partly due to the significant male predominance of the patients included in this review. Similarly, the literature on ACLR is still predominantly related to the male gender; however, this is shifting rapidly, and the considerations of female ACLR should be considered high on the agenda for future research priorities in soft tissue knee surgery [24–26].

Height, weight, thigh length and circumference all demonstrated a moderately positive correlation with graft size within this review. Such anthropomorphic measurements can be considered surrogate markers for muscular development, both in relation to cross-sectional area and axial muscular length and thus can be considered more relevant markers to base potential graft size upon. On the other hand, BMI demonstrated a weak correlation with graft size, supporting the notion that lean body mass calculation should be used in favour of BMI when considering eventual graft size, as reported in studies by Abatsi et al. [22, 27].

### Graft subgroup analysis

PLT-using studies demonstrated a strong correlation with height, weight, and graft diameter in comparison to hamstring-using studies. The reasons for this have not been born out in the literature but may support the notion that utilising the PLT as a graft of choice may have more reproducible and reliable clinical results if the treating clinician relies on anthropomorphic measurements in the pre-operative phase. However, to further validate these clinical conclusions, standardised methods of graft sizing and reporting would be required, and heterogeneity in their reporting within the context of this study may discredit any conclusions that can be drawn relating to the utility of different graft types.

### Limitations

Anthropometric data should be used contextually, with generalisability not applicable between differing populations. For example, specific data relating to graft thickness in Caucasian populations may not correlate with recommendations for patients in South East Asia due to genetic differences in musculoskeletal structure between different populations[28]. This review included data from various populations with subanalysis performed based on various regions; however, the skew was towards the Indian and American populations. Further work should generalise the analysis with equal representations from different populations.

This review predominantly focused on ACLR in the male population, with 94.8% of included patients male. Within ACLR, female patients experience high rates of graft–tunnel mismatch, laxity and re-rupture than male patients [29]. This furthers the notion that future research into the female population is critical, with research into graft choice and reasons for failure high on the agenda for practising clinicians. Work to address the limitations of this systematic review may be best addressed by considering the routine and widespread implementation of registries for ACLR. This should focus on standardised sizing criteria for grafts and utilising comparable outcome measurements. By facilitating access to outcome information for ACLR, evidenced-based decisions relating to suitability for surgery, graft choice, and the outcome would ultimately improve patient outcomes.

As surgeons gain more confidence in selecting appropriate graft types and planning surgeries based on anthropometric measurements, it could lead to better surgical outcomes. This, in turn, could contribute to reduced reoperation rates and healthcare costs, which may have implications for public health resource allocation. Also, improved pre-operative planning and graft size selection could potentially lead to fewer post-operative complications and revisions. This could alleviate the burden on the healthcare system, allowing resources to be directed towards other pressing health issues.

## Conclusion

This study demonstrated a significant moderately positive correlation between anthropometric measurements (height, weight, thigh circumference, and length) and ACL graft size, a significant weak positive correlation with BMI, and an insignificant correlation for age and gender. Height was more strongly correlated with graft diameter in the peroneus longus tendon than hamstring grafts. These findings can assist in selecting the appropriate graft size for ACL reconstruction based on patient anthropometric data.

### Supplementary Information

Below is the link to the electronic supplementary material.

Supplementary file 1 (DOCX 375 kb)

## Data Availability

Available upon request.
