# Supplementary material for: Correlation between anthropometric measurements and graft size in anterior cruciate ligament reconstruction: a systematic review and meta-analysis
Source: Eur J Orthop Surg Traumatol. 2023 Sep 6;34(1):97–112. doi: 10.1007/s00590-023-03712-w (PMC10771386; doi:10.1007/s00590-023-03712-w)
Supplement: Supplementary file 1 — (DOCX 375 kb) [file 590_2023_3712_MOESM1_ESM.docx]

**Supplementary material**

**Table 1.** MINORS criteria.

| **Study/Items** | **A clearly stated aim** | **Inclusion of Consecutive patients** | **Prospective collection of Data** | **Endpoints appropriate to the aim of the study** | **Unbiased assessment of the study endpoints** | **Follow-up period appropriate to the aim of the study** | **Loss to follow up less than 5%** | **Prospective calculation of the study size** | **Total** |
| --- | --- | --- | --- | --- | --- | --- | --- | --- | --- |
| 2007 Brown | 2 | 2 | 2 | 2 | 2 | 2 | 2 | 0 | 14 |
| 2007 Tuman | 2 | 2 | 2 | 2 | 2 | 2 | 2 | 0 | 14 |
| 2008 Treme | 1 | 2 | 2 | 2 | 2 | 2 | 2 | 0 | 13 |
| 2012 Chan | 2 | 2 | 2 | 2 | 2 | 2 | 2 | 0 | 14 |
| 2012 Reboonlap | 2 | 2 | 2 | 2 | 2 | 2 | 2 | 0 | 14 |
| 2012 Stergios | 2 | 2 | 0 | 2 | 2 | 2 | 2 | 0 | 12 |
| 2012 Xie | 2 | 0 | 2 | 2 | 2 | 2 | 2 | 0 | 12 |
| 2013 Celiktas | 2 | 2 | 2 | 2 | 2 | 2 | 2 | 0 | 14 |
| 2013 Challa | 2 | 2 | 2 | 2 | 2 | 2 | 2 | 0 | 14 |
| 2013 Park | 2 | 0 | 0 | 2 | 2 | 2 | 2 | 0 | 10 |
| 2013 Thomas | 2 | 2 | 0 | 2 | 2 | 2 | 0 | 0 | 10 |
| 2014 Schwartzberg | 2 | 2 | 2 | 2 | 2 | 2 | 2 | 0 | 14 |
| 2015 Nuelle | 2 | 2 | 2 | 2 | 2 | 2 | 2 | 0 | 14 |
| 2016 Asif | 1 | 2 | 2 | 2 | 2 | 2 | 2 | 0 | 13 |
| 2016 Atbasi | 2 | 2 | 2 | 2 | 2 | 2 | 2 | 0 | 14 |
| 2016 Calvo | 2 | 2 | 2 | 2 | 2 | 2 | 2 | 0 | 14 |
| 2016 Goyal | 2 | 2 | 0 | 2 | 2 | 2 | 2 | 0 | 12 |
| 2016 Ho | 2 | 0 | 2 | 2 | 2 | 2 | 2 | 0 | 12 |
| 2016 Kivi | 2 | 2 | 2 | 2 | 2 | 2 | 2 | 0 | 14 |
| 2016 Pereira | 2 | 2 | 2 | 2 | 2 | 2 | 2 | 0 | 14 |
| 2016 Sundararajab | 2 | 0 | 0 | 2 | 2 | 2 | 2 | 0 | 10 |
| 2017 Chiba | 2 | 2 | 0 | 2 | 2 | 2 | 0 | 0 | 13 |
| 2017 Gupta | 2 | 1 | 2 | 2 | 2 | 2 | 2 | 0 | 13 |
| 2017 Leiter | 2 | 1 | 0 | 2 | 2 | 2 | 2 | 0 | 11 |
| 2017 Vincent V.G. An | 2 | 1 | 0 | 2 | 2 | 2 | 2 | 0 | 11 |
| 2018 Corey | 2 | 1 | 0 | 2 | 2 | 2 | 2 | 0 | 11 |
| 2018 Ramkumar | 2 | 1 | 0 | 2 | 2 | 2 | 2 | 0 | 11 |
| 2018 Song | 2 | 2 | 0 | 2 | 2 | 2 | 2 | 0 | 12 |
| 2019 Heijnoer | 2 | 1 | 2 | 2 | 2 | 2 | 2 | 0 | 13 |
| 2019 Moghamis | 2 | 2 | 2 | 2 | 2 | 2 | 2 | 0 | 14 |
| 2019 Rhatomy | 2 | 2 | 0 | 2 | 2 | 2 | 2 | 0 | 12 |
| 2019 Sakti | 2 | 2 | 2 | 2 | 2 | 2 | 2 | 0 | 14 |
| 2020 Du-Hyun Ro | 2 | 1 | 0 | 2 | 2 | 2 | 2 | 1 | 12 |
| 2020 Goyal | 2 | 1 | 2 | 2 | 2 | 2 | 2 | 0 | 13 |
| 2020 Jagadeesh | 2 | 1 | 2 | 2 | 2 | 2 | 2 | 0 | 13 |
| 2020 sakti | 2 | 1 | 2 | 2 | 2 | 2 | 2 | 0 | 13 |
| 2020 Thwin | 2 | 2 | 0 | 2 | 2 | 2 | 2 | 0 | 12 |
| 2021 Ertilav | 2 | 1 | 0 | 2 | 2 | 2 | 2 | 0 | 11 |
| 2021 Gagliardi | 2 | 1 | 0 | 2 | 2 | 2 | 2 | 0 | 11 |
| 2021 Khan | 2 | 1 | 0 | 2 | 2 | 2 | 2 | 0 | 11 |
| 2021 Kumar | 2 | 1 | 2 | 2 | 2 | 2 | 2 | 0 | 13 |
| 2021 Singhal | 2 | 1 | 2 | 2 | 2 | 2 | 2 | 2 | 15 |
| 2022 Harshith.R | 2 | 1 | 2 | 2 | 2 | 2 | 2 | 0 | 13 |
| 2022 Huang | 2 | 1 | 1 | 2 | 2 | 2 | 2 | 0 | 12 |
| 2022 Mishra | 2 | 1 | 2 | 2 | 2 | 2 | 2 | 0 | 13 |
| 2023 Movahedinia | 2 | 1 | 2 | 2 | 2 | 2 | 2 | 0 | 13 |

**Forest plots of the Subanalysis based on region**

**Funnel plots for all pooled correlations:**

**Sensitivity analysis which removed all small studies (n <100):**
